# Reactive Oxygen Species and Nitric Oxide in Cutaneous Leishmaniasis

**DOI:** 10.1155/2012/203818

**Published:** 2012-04-12

**Authors:** Maria Fátima Horta, Bárbara Pinheiro Mendes, Eric Henrique Roma, Fátima Soares Motta Noronha, Juan Pereira Macêdo, Luciana Souza Oliveira, Myrian Morato Duarte, Leda Quercia Vieira

**Affiliations:** ^1^Departamento de Bioquímica e Imunologia, Instituto de Ciências Biológicas, Universidade Federal de Minas Gerais, 31270-901 Belo Horizonte, MG, Brazil; ^2^Departamento de Microbiologia, Instituto de Ciências Biológicas, Universidade Federal de Minas Gerais, 31270-901 Belo Horizonte, MG, Brazil; ^3^Núcleo de Pesquisas em Ciências Biológicas (NUPEB), Instituto de Ciências Biológicas e Exatas, Universidade Federal de Ouro Preto, Morro do Cruzeiro, 35400-000 Ouro Preto, MG, Brazil

## Abstract

Cutaneous leishmaniasis affects millions of people around the world. Several species of *Leishmania* infect mouse strains, and murine models closely reproduce the cutaneous lesions caused by the parasite in humans. Mouse models have enabled studies on the pathogenesis and effector mechanisms of host resistance to infection. Here, we review the role of nitric oxide (NO), reactive oxygen species (ROS), and peroxynitrite (ONOO^−^) in the control of parasites by macrophages, which are both the host cells and the effector cells. We also discuss the role of neutrophil-derived oxygen and nitrogen reactive species during infection with *Leishmania*. We emphasize the role of these cells in the outcome of leishmaniasis early after infection, before the adaptive T_h_-cell immune response.

## 1. Introduction

More than 20 *Leishmania* species cause leishmaniasis in people with different genetic backgrounds and general states of health. Further, the diversity of clinical manifestations, epidemiology, and immunopathology makes leishmaniasis a complex disease to study. Clinical manifestations include ulcerative skin lesions, destructive mucosal inflammation, and disseminated visceral infection (kala azar). Morbidity includes disfigurement and disability. However, some features are shared by all forms of infection by these protozoan parasites: parasitism is persistent, tissue macrophages are the main parasitized cell, and the host immune response defines the outcome of the disease [1].

Cutaneous leishmaniasis is caused by several species of the genus *Leishmania*, including *L. major, L. tropica, L. aethiopica, L. mexicana, L. braziliensis, L. guyanensis, L. panamensis, L. peruviana*, and* L. amazonensis. *The *Leishmania *genus is divided in two subgenera, *Leishmania *and *Viannia.* In the subgenus *Leishmania, L. amazonensis*, *L. mexicana *(complex *L. mexicana*), and *L. major *(complex *L. major*) are by far the most studied species that cause cutaneous leishmaniasis. The subgenus *Viannia *comprises two important species that cause cutaneous leishmaniasis, *L. guyanensis *(complex *L. guyanensis*) and *L. braziliensis* (complex *L. braziliensis*) [2, 3].

The promastigote stage of the parasite lives in the gut of sandflies (*Phlebotomus *in the Old World and* Lutzomyia* in the New World) [4]. In the insect gut, *Leishmania* promastigotes develop into metacyclic (infective) forms and enter the vertebrate host when female sandflies take a blood meal. In the vertebrate host, phagocytic cells ingest the metacyclic promastigotes that, inside the phagolysosome, differentiate into the amastigote form and replicate. The amastigotes rupture the macrophage and proceed to infect other macrophages in the tissue, and, if unchecked by the immune system, they will replicate indefinitely. The parasites rely on macrophages for successful replication, although they can also be taken up by neutrophils [5, 6] and dendritic cells [7]. *Leishmania *do not enter cells actively; thus, they are macrophage obligatory parasites, and the mechanism of entrance is accepted to be phagocytosis [7]. The exit of parasites from the macrophage is less clear. It is becoming apparent that the release of intracellular pathogens is not simply a consequence of a physical or metabolic burden imposed on the host cell, but rather of particular exit strategies governed by the microorganisms (reviewed in [8]). In *Leishmania*, parasite*-*derived pore-forming cytolysins, which we call leishporin, may be involved [8–13]. The life cycle of *Leishmania* is complete when sandflies feed on infected hosts, ingesting infected cells.

Although the immune response induced by infection with *Leishmania* has been the subject of many investigations, the mechanisms that underlie host resistance and pathogenesis in leishmaniasis are not entirely understood. During the late 80s and early 90s, the discovery of two distinct subpopulations of CD4+ T helper cells based on their cytokine production, Th1 and Th2 [14], finally explained resistance and susceptibility to *L. major* in the murine model. The resistance of C57BL/6 and the susceptibility of BALB/c mice were shown to be the result of the development of a Th1 or Th2 response, respectively. IFN-*γ* produced by Th1 cells induces the expression of inducible nitric oxide synthase (iNOS or NOS2) by macrophages. This enzyme catalyzes the oxidation of the guanidino nitrogen of l-arginine to produce nitric oxide (NO), which kills the parasite. In contrast, the Th2 response not only activates macrophages to produce arginase (by the action of IL-4, IL-13, and IL-10), which competes with iNOS for the same substrate, but also inhibits the ability to produce NO [15–19]. For some time, Th1 cells and NO were thought to be the sole protagonists of mouse resistance to leishmaniasis, until other reports (referred below) showed that the polarization of the response to Th1 or to Th2 does not explain host resistance or susceptibility to all species of *Leishmania* and does not occur in all host/parasite combinations. Hence, infection with *L. amazonensis* is an example of the still controversial nature of protective immunity in mice. The disease caused in C57BL/6 mice by *L. amazonensis*, for instance, appears to depend on Th1 cells [20], and lesions in C3HeB/FeJ mice do not heal after induction of a Th1 response during chronic infection [21]. However, Th1 cells help mice control *L. amazonensis* infection established by promastigotes, but not by amastigotes [22], and a Th1 response elicited by *L. major* confers resistance in C3HeB/FeJ and C57BL/6 mice to *L. amazonensis *challenge [23, 24]. Likewise, the lack of resistance of C57BL/10 to *L. amazonensis *infection [25] and of BALB/c to *L. mexicana *[26] does not correlate with the presence of a typical Th2 response, suggesting that susceptibility to these species of *Leishmania* is due to a failure to mount a Th1 response, rather than the presence of a Th2 response. Conversely, the resistance of BALB/c to *L. braziliensis *appears to be due to the absence of a Th2 response rather than to the presence of a Th1 response [27]. The inconsistency of the pattern protection/Th1 and pathogenesis/Th2 to all species of *Leishmania *was recently reviewed [28].

 Indeed, except for a few references [29–31], innate immunity has largely been overlooked with respect to the mechanism of host resistance to *Leishmania* infection. Dendritic cells, macrophages, and neutrophils, along with their early-produced cytokines and reactive nitrogen and oxygen species, have not been spotlighted as effector cells during the initial stages of infection. Even the leishmanicidal competence of macrophages has mostly been described as a T-cell-dependent event, even though inducers of NO are available very early after infection, namely, type 1 interferons (IFN-**α** and IFN-**β**) and type 2 (IFN-*γ*) interferons. While IFNs-**α** and -**β** have been shown to be secreted by macrophages [32], IFN-*γ* is produced by NK cells [16, 30, 33, 34] and possibly by *γ*/*δ* T cells [35], NKT cells [35], or even macrophages [36, 37], although the latter is still controversial [38]. More recently, however, innate immunity effector cells have been suggested to be coparticipants in the maintenance or elimination of the parasites, acting in the early stages of infection in the absence of a T_h_-cell response.

In this paper, we highlight the participation of both NO and reactive oxygen species (ROS) in the resistance and pathogenesis of cutaneous leishmaniasis. We first address the fate of promastigotes in the initial phase of the infection, discussing the role of these leishmanicidal molecules in eliminating part of the parasite burden while the adaptive response is still absent (innate immunity). We also discuss the role of these molecules at later phases of the disease, when T_h_ cells are available (adaptive immunity). In both circumstances, we emphasize the differences among the various *Leishmania* species and mouse strains. The mechanisms that *Leishmania *utilize to evade killing by NO and ROS have been the subject of a recent review and will not be discussed here [39].

## 2. ROS and NO

Neutrophils and macrophages produce ROS in response to phagocytosis and ligands of pattern recognition receptors (PRRs). The patterns recognized by PRRs can be either of pathogenic origin (pathogen-associated molecular patterns (PAMPs)) or induced by danger patterns (damage-associated molecular patterns (DAMPs)) that signal tissue damage, which are generally hidden from PRRs, such as ATP [40–42]. Moreover, endothelial activation can also induce ROS production by neutrophils [43]. In response to these signals, nicotinamide adenine dinucleotide phosphate- (NADPH-) dependent phagocyte oxidase (Nox2, also known as phox or gp91*^phox^*) is assembled, and superoxide is produced from molecular oxygen [44, 45]. Superoxide may be dismutated into hydrogen peroxide, which can, in turn, generate hydroxyl radicals and other ROS. Macrophages produce ROS in higher quantities than neutrophils [43, 46, 47].

NO is also produced by neutrophils and macrophages in response to IFN-*γ* and a second signal provided by a PAMP ligand or TNF-**α**. iNOS expression is induced by these signals. iNOS promotes the oxidation of the guanidino nitrogen of l-arginine, resulting in the production of NO and citrulline [47].

In activated macrophages, superoxide and NO are produced in nearly equimolar quantities and generate peroxynitrite (ONOO^−^), a free radical that is also highly toxic to pathogens [48].

## 3. First Encounters—The Neutrophils

As early as 30 seconds after exposure of C57BL/6 mice to *L. major* through the bite of infected sandflies or needle inoculation of promastigotes, the injected area is infiltrated by neutrophils, which has been elegantly visualized by two-photon intravital microscopy [49]. Recruited neutrophils readily phagocytose promastigotes, which remain viable, although it is not known to what extent parasites are taken up or survive. In fact, it has been reported that during the first 24 h, most parasites are localized extracellularly and can be taken up later by macrophages [49]. The above report showed that parasites taken up by the early neutrophil migration are kept alive inside these cells and do not suffer from oxidative stress. However, another study showed that at later time points, neutrophils might play a role in parasite attrition [50], and, within 2 days, parasites inside neutrophils show a wide variation in their morphology from healthy to completely destroyed forms [50]. Killing of intracellular parasites has been identified by severe signs of damage, such as aggregated cytoplasm and extended vacuolization or complete lysis [50], indicating that neutrophils can act as parasite killers within the first few days of infection. Neutrophils act through an array of microbicidal mechanisms, of which the ability to produce NO [51] and ROS [52] are the most studied in leishmaniasis. Indeed, *L. major* has been shown to induce NO production by mouse neutrophils *in vitro* [53] and to stimulate the respiratory burst in mouse [54], rabbit [55], and human [56] neutrophils. Another study, however, showed that *L. major *failed to induce a respiratory burst in human neutrophils, and *L. major-*containing phagosomes did not colocalize with granules involved in superoxide production [57]. However, work by Peters et al. [49] has very eloquently shown that there is no oxidative stress within the first hours of infection.

Inflammatory neutrophils harvested from BALB/c mice four hours after i.p. infection with *L. major *harbor more parasites than C57BL/6 cells, which, in turn, produce considerably higher amounts of NO than BALB/c in response to *L. major* and IFN-*γ* [53]. In agreement with these data, we have shown that neutrophils from uninfected C57BL/6 mice express much more iNOS and produce more NO than cells from BALB/c mice when stimulated with IFN-*γin vitro*, indicating that the ability of these cells to be activated to produce NO is inherent to each strain. These data suggest that NO produced by neutrophils may help to control infection with *L. major* in very early disease stages. *In vitro*, however, iNOS expression and NO production can be inhibited in neutrophils from both mouse strains by live, but not dead, promastigotes of *L. major* (our unpublished results).

In BALB/c mice, an iron-induced oxidative burst appears to prevent the growth of *L. major*, protecting the animals from developing the typical large lesions. This oxidative burst has mainly been attributed to neutrophils [58, 59]. However, C57BL/6 resistance and BALB/c susceptibility inversely correlate with the ability of their neutrophils to generate ROS since BALB/c neutrophils produce more ROS than C57BL/6 neutrophils when stimulated with phorbol myristate acetate (PMA). *L. major* has also been shown to inhibit a PMA-induced respiratory burst in neutrophils from both strains of mice (our unpublished results).

Interestingly, the rapid recruitment of neutrophils to *L. major*-induced lesions was previously reported to follow different kinetics in susceptible BALB/c and resistant C57BL/6 mice, which might account for these opposite outcomes. In susceptible mice, almost 100% of the initial cellular infiltrate is composed of neutrophils, half of which is replaced by mononuclear phagocytes in 2-3 days. Neutrophils comprise the other half of the cellular infiltrate for at least 12 days after infection. In contrast, in resistant mice, only about 60% of the initial cellular infiltrate is composed of neutrophils, and the number of these cells drastically decreases to only 1-2% at later time points. In resistant mice, mononuclear phagocytes predominate at later time points, comprising more than 70–80% of the cells [49]. Notably, infection with *L. major* also results in the differentiation of distinct neutrophil populations in BALB/c and C57BL/6 mice. The parasite induces CD49d expression in BALB/c, but not in C57BL/6, neutrophils. The levels of Toll-like receptor (TLR) 2, TLR7, and TLR9 mRNA are significantly higher in C57BL/6 cells than in BALB/c cells. Moreover, C57BL/6, but not BALB/c, neutrophils secrete biologically active IL-12p70 and IL-10. BALB/c neutrophils instead transcribe and secrete high levels of IL-12p40, which forms homodimers with inhibitory activity. In C57BL/6 mice, neutrophils may constitute one of the earliest sources of IL-12, while in BALB/c mice, secretion of IL-12p40 may contribute to impaired early IL-12 signaling [53]. Furthermore, C57BL/6 neutrophils were found to release 2-3-fold more elastase than BALB/c cells, which contributes to parasite killing through activation of TLR4 [60]. These distinct neutrophil phenotypes may thus influence both the early resistance or susceptibility and the development of an *L. major*-specific immune response. The role of these different populations of neutrophils on resistance to parasites through reactive nitrogen and oxygen species production deserves further investigation.

Recently, the interaction of neutrophils and macrophages has been investigated *in vitro* (reviewed in [5]). Dead neutrophils from C57BL/6 mice can activate infected macrophages to kill *L. major*. In this system, activation is mediated by the induction of TNF-*α* by neutrophil elastase, but NO is not involved in parasite killing. Rather, superoxide is partially responsible for parasite killing, as evidenced by the partial inhibition of this effect when catalase was added to this *in vitro *system [60, 61]. The same results were obtained with dead human neutrophils and *L. amazonensis*-infected human macrophages [62]. In another study, live murine neutrophils induced killing of *L. braziliensis*, but not *L. major*, by infected macrophages. Superoxide production was detected in this system, and killing of parasites was inhibited by *N*-acetylcysteine, a superoxide scavenger. Killing of *L. braziliensis* by macrophages cocultured with live neutrophils was also independent of NO [63]. Neutrophil-induced killing of *L. amazonensis *by macrophages from resistant and susceptible mouse strains was also described and is mediated by neutrophil elastase, TNF-*α*, and platelet-activating factor (PAF), but not by NO or reactive oxygen species [64].

 In response to pathogens, neutrophils may release the so-called neutrophil extracellular traps (NETs), which are fibrous nets composed of decondensed chromatin, histones, and granule antimicrobial proteins that trap and kill microbes extracellularly [65, 66]. NETs extruded by human neutrophils cultured *in vitro* were shown to kill *L. amazonensis, L. major*, and *L. chagasi*. These NETs were found in lesions from patients. Killing of parasites was found to be mediated mainly by histones [67]. Importantly, NET formation is defective in patients suffering from chronic granulomatous disease, who lack Nox2 activity [68]. In fact, reactive oxygen species are required to initiate NETs. Oxidative stress ruptures neutrophil elastase and mieloperoxidase-containing granules, and neutrophil elastase binds to chromatin and cleaves histones, a reaction that is further enhanced by mieloperoxidase, independent of its enzymatic activity. This enzyme promotes chromatin decondensation, which culminates in NET release due to cellular rupture [69]. The molecular mechanism linking ROS production to chromatin decondensation and binding to antimicrobial proteins is still unknown.

Although several *in vivo* studies have addressed the role of neutrophils during infection with *L. major*, their function in resistance to the parasite is not totally understood and is still a subject of debate. Due to the heterogeneous models used to study the role of neutrophils in experimental leishmaniasis, it is still unknown whether these cells have a protective or pathogenic role. Like other immune responses in murine models, the neutrophil function appears to depend on the species and even the strain of *Leishmania* and the genetic background of mice used as host (thoroughly reviewed in [70]). Hence, even less clear is the *in vivo *role of reactive oxygen and nitrogen species from neutrophils in *Leishmania *resistance or pathology caused by the parasites. However, *in vitro *evidence suggests that ROS from neutrophils are involved in killing of the parasite, suggesting that ROS may be important for resistance to parasites early in infection.

## 4. Latecomers—The Macrophages

Like neutrophils, macrophages are microbicidal cells that are able to produce NO and ROS [47]. Paradoxically, these cells are also the long-term host cell for *Leishmania*. In experimental leishmaniasis, macrophages are as crucial for parasite survival as for its elimination [71]. The role played by these cells depends on the type of activation and the vulnerability of the parasite to the effector mechanisms.

The mechanism by which macrophages are responsible for resistance to *Leishmania *was first characterized by *in vitro *experiments using murine macrophages infected with *L. major*. In this model, killing of parasites is dependent on the activation of macrophages by IFN-*γ* and a second signal that triggers TNF-*α*. This signal is given by amastigotes, promastigotes, or parasite-derived glycoinositolphospholipids (GIPLs) and lipophosphoglycan (LPG), but not by killed cells or cellular lysates. Once these two signals are present, iNOS is induced and NO is produced [72–74]. The clear role of NO in killing *L. major *was established by pharmacological inhibition of the production of NO *in vitro* and by the observation of a higher susceptibility of iNOS knockout mice to infections with *L. major* [16, 74–76]. It was further confirmed by the inability of macrophages from iNOS knockout mice to be activated and kill *L. major *by IFN-*γ* [77]. Hence, NO clearly has a crucial role in killing of *L. major* by IFN-*γ*-activated macrophages.

**Figure 1:**
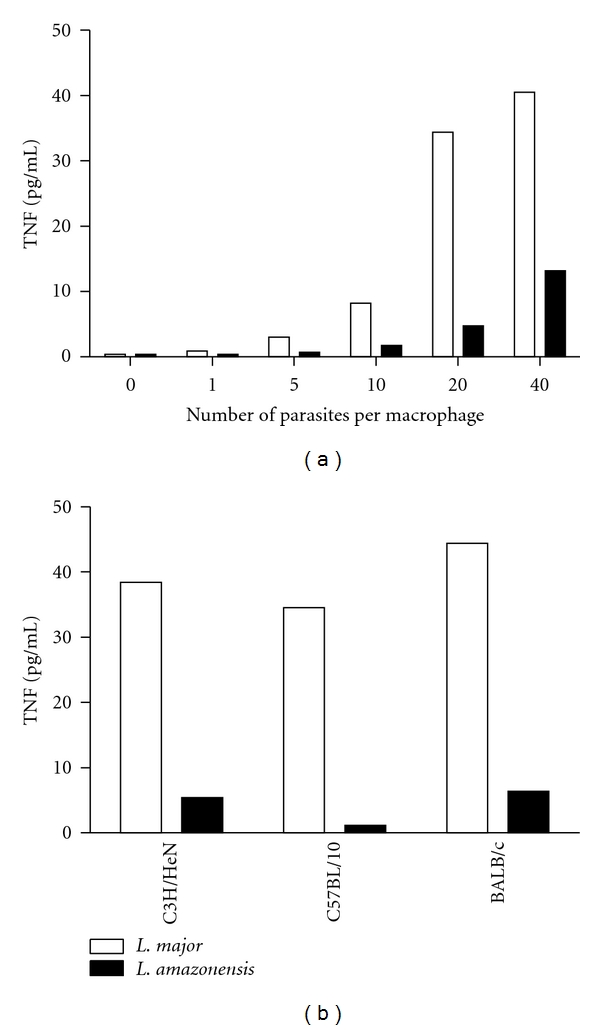
Infection with *L. major *induces more TNF than infection with *L. amazonensis. *(a) TNF production by inflammatory macrophages from C57BL/10, mice infected *in vitro *with *L. major *or *L. amazonensis. *(b) Production of TNF *ex vivo *by lymph node cells from C3H/HeN, C57BL/10 and BALB/c mice infected with *L*. *major *or *L. amazonensis*, 2 days after infection. A biological assay that does not distinguish between TNF-*α* or TNF-*β* was used in these experiments. These are representative experiments of more than five performed experiments (L. Q. Vieira and P. Scott, unpublished).

During *L. amazonensis* infection, IFN-*γ* and TNF-*α* are not produced at high levels as in *L. major *infection [25, 78]. Therefore, infection of *L. major*-resistant mice with *L. amazonensis* leads to chronic lesions and inefficient control of parasites at the site of infection. IFN-*γ*-activated macrophages from CBA/J mice infected with either *L. major *or *L. amazonensis* are able to kill the former, but not the latter. When very high concentrations of NO were generated *in vitro*, axenic *L. amazonensis* amastigotes succumbed. In addition, macrophages infected with *L. amazonensis *produce less TNF-*α* when compared to those infected with *L. major *[79]. However, macrophages infected with either *L. major *or *L. amazonensis *produce similar levels of NO (measured as nitrite in culture supernatants) and express similar levels of iNOS message when activated with IFN-*γ* [79]. Corroborating these data, we found lower levels of TNF (**α** and **β** were measured collectively) from *L. amazonensis*-infected macrophages from C57BL/10 mice than from *L. major*-infected macrophages (Figure 1(a)). In addition, two days after infection in the hind footpad, popliteal lymph node cells from C3H/HeN, C57BL/10 (mouse strains resistant to *L. major*), and BALB/c mice produced more TNF *ex vivo *when infected with *L. major *than with *L. amazonensis *(Figure 1(b)). Interestingly, *L. amazonensis-*infected CBA/J macrophages also produce less reactive oxygen species than *L. major*-infected cells [79], which could be, in part, responsible for the different abilities of macrophages to kill these two species of *Leishmania*. The mechanism by which *L. amazonensis *resists killing remains unknown.

Even more intriguing is the observation that low doses of IFN-*γ* actually promote amastigote growth within macrophages [22]. In accordance with this observation, at later stages of infection, increased amounts of NO were found in the more susceptible BALB/c mice than in C57BL/6 mice infected with *L. amazonensis* as lesions progressed and parasites expanded because C57BL/6 mice partially control lesions and parasite growth [80].

IFN-*γ*-activated macrophages represent the host-parasite interaction in which T cells are already producing a large amount of this cytokine. During the first 2 days after infection with *L. major*, nearly all macrophages recruited to the site of infection contain phagocytosed parasites, both in C57BL/6 and in BALB/c mice. However, the percentage of cells (mostly neutrophils and mononuclear phagocytes) containing intact parasites in BALB/c mice is higher than that in C57BL/6 cells (mostly mononuclear cells), and the elimination of parasites from the site of infection is higher in resistant mice [50]. This suggests that parasites may also be killed by tissue mononuclear cells well before the onset of a T-cell response. Whether this killing is mediated by reactive oxygen and nitrogen species remains unknown. 

Isolated macrophages from C57BL/6 mice produce more NO than macrophages from susceptible strains when stimulated with IFN-*γ* [81–84], TNF-*α* [81, 85], or LPS [83, 85–89]. This is an interesting but poorly explored aspect of the murine models of resistance/susceptibility to microbial infections, which is clearly independent of the development of an adaptive Th1 or Th2 response. Mills et al. [90] systematically tested this observation and generalized it to other strains of mice. They showed that macrophages from strains that are typical Th1 responders (termed M-1) or typical Th2 responders (termed M-2) differ qualitatively in their ability to be activated, as measured by their arginine metabolic programs. M-2 macrophages from BALB/c mice (prototypes of Th2 responders) stimulated with a particular concentration of LPS not only produce little or no NO, but increase arginine metabolism to ornithine. In contrast, M-1 cells from C57BL/6 mice (prototypes of Th1 responders) generate a strong NO and citrulline response and appear to decrease their production of ornithine.

We investigated the molecular basis of the differential production of NO by macrophages from mice with resistant or susceptible phenotypes to *L. major* by *in vitro *stimulation with IFN-*γ* and LPS. We have shown that M-1 macrophages show a remarkably strong expression of the enzyme iNOS upon stimulation when compared to M-2 cells [84]. The accumulation of iNOS mRNA is also higher in M-1 cells. Interestingly, however, we found that the accumulation of the iNOS protein is more dramatic than the accumulation of iNOS mRNA. The accumulation of both iNOS mRNA and protein is not a consequence of a higher stability of the molecule. The data showed that iNOS gene expression is differentially regulated in M-1 and M-2 macrophages and suggested that it is transcribed and translated at different rates in these two types of cells [84]. Recent results from our group indicate that the higher iNOS expression in M-1 macrophages may be multifactorial and may be regulated by higher levels of TNF-*α*, IL-12, and IFN-*β* (unpublished data).

The intrinsic differential sensitivity to IFN-*γ* and LPS of M-1 or M-2 cells has led to two important observations regarding the *in vivo* infection.

(1) Small amounts of IFN-*γ* (from NK, NKT, or *γ*/*δ* T cells) or other pathogen-derived inducers may induce M-1, but not M-2 cells, to kill the pathogen through NO, before T cells differentiate into the IFN-*γ*-Th1 subpopulation. In fact, larger numbers of *L. major *are found in iNOS-deficient macrophages than in wild-type macrophages 72 hours after infection, indicating that some NO is produced by macrophages that have not been activated with IFN-*γ* and that NO, even if not detectable, exerts some control of parasite growth [75, 77]. Further evidence of a NO-dependent T_h_-cell-independent mechanism was obtained when resting human macrophages were infected with NO-susceptible and NO-resistant *L. amazonensis *and *L. braziliensis* isolates and selected *in vitro *with increasing concentrations of NaNO_2_: NO-resistant parasites grew better in resting macrophages than the NO-susceptible isolates [91].

(2) Activated M-1 and M-2 cells can distinctly affect subsequent production of Th1-dominant or Th2-dominant cytokines (IFN-*γ* or TGF-**β**1, resp.), positioning macrophages as key performers in directing the Th1 or Th2 outcome. M-1 and M-2 macrophages differentially influence the Th lymphocyte response, and how macrophages are stimulated determines the route that Th responses will take [90]. These observations indicate that macrophages may contribute to the outcome of an immune response through mechanisms other than by acting as established NO-producing cells and that their role in determining the resistant/susceptible phenotype in mice may be significant. M-1 macrophages not only can mount an early (innate) resistance, but also can consolidate the status of resistance by favoring a Th1 adaptive response.

In addition to NO, ROS are considered to be a major macrophage effector mechanism induced by IFN-*γ* to control infections. Upon bacteria or other pathogen engulfment by a phagocytic cell, ROS are rapidly produced by NADPH oxidase, an enzymatic complex comprised of membrane bound (p22*^phox^* and gp91*^phox^*) and cytosolic (p40*^phox^*, p47*^phox^*, p67*^phox^*, and Rac-1/2) proteins [45, 92], which may be assembled after TLR stimulation by bacterial products via MyD88-dependent p38 MAPK activation [93].

**Figure 2:**
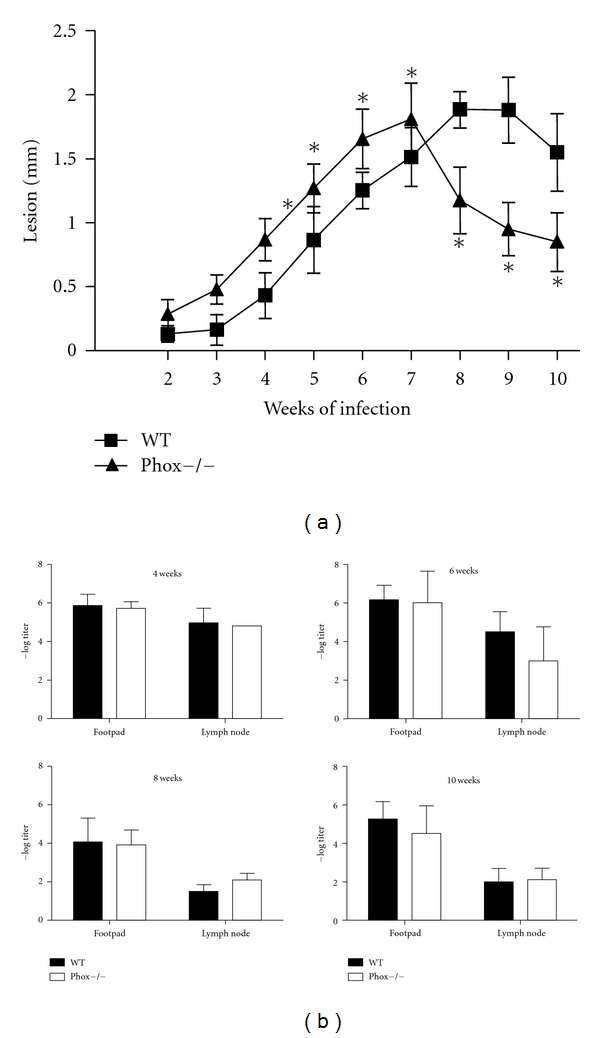
Course of infection with *L. amazonensis* in wild-type C57BL/6 and Nox2 knockout mice (a) and parasite quantitation using a limiting dilution analysis (b). *indicates statistical difference by Student's *t* test, *P* < 0.05 (E. H. Roma and J. P. Macedo, unpublished).

 Macrophages [54, 76] and neutrophils [54] produce ROS in response to* Leishmania in vitro*. Killing of *L. major* by IFN-*γ*-activated macrophages is dependent on NO production, but not on the production of superoxide or peroxynitrite [76]. Lesions in Nox2 knockout mice [94] (Nox2 mice are genetically deficient in the NADPH-dependent phagocyte oxidase. These mice were originally described as a model for chronic granulomatous disease and are more susceptible to bacterial infection, and neither neutrophils nor macrophages present respiratory burst oxidase activity [94].) infected with *L. major* are similar to those in wild-type C57BL6 mice. Nox2 knockout mice control *L. major* at the site of infection at early time points, but display an unexpected reactivation of *L. major* infection after long periods of observation (more than 200 days of infection). Further, they show deficient control of parasite replication in draining lymph nodes and spleens, suggesting that Nox2 is important for the control of *L. major in vivo* at later times of infection by preventing visceralization [54]. The participation of ROS in killing of *L. amazonensis* by mouse [95, 96] or human [97] macrophages has been reported. Our preliminary data suggest that macrophages from Nox2 knockout mice behave similarly to macrophages from wild-type mice when infected with *L. amazonensis. *Moreover, similar to infection with *L. major*, Nox2 knockout mice control parasites at the site of infection as well as wild-type mice (Figure 2). Surprisingly, at earlier times of infection, lesions are larger in Nox2 knockout mice, and, at later times of infection, they become smaller than in wild-type mice (Figure 2(a)). This indicates that the differences in Ros activity on macrophage behavior at different stages of infection may be due to differences in the inflammatory infiltrate. The contradictions between the *in vitro* evidence for a role for ROS in resistance to *L. amazonensis *and *in vivo *data remain to be explained.

Although BALB/c mice are the prototype model of susceptibility to most species of *Leishmania *(such as *L. major* and *L. amazonensis*), *L. braziliensis* [27, 98] and *L. guyanensis* [99] do not cause large skin lesions in this mouse strain. Our studies using *L. guyanensis* have shown that BALB/c mice develop minor or no lesions, do not enable parasite replication, and do not die of the infection. In addition, *L. guyanensis* [99] and *L. braziliensis* [100], unlike *L. amazonensis*, fail to survive within nonactivated peritoneal macrophages *in vitro*. *In vitro* infection of BALB/c macrophages with *L. guyanensis* does not activate the production of NO; instead, it activates a respiratory burst that is exceptionally higher than that activated by infection with *L. amazonensis. *We have further shown that the production of ROS is responsible for the elimination of *L. guyanensis* by macrophages. We have also shown that *L. guyanensis* amastigotes die inside BALB/c macrophages through an apoptosis-like process mediated by parasite-induced ROS [99]. These findings demonstrate an important killing mechanism of *L. guyanensis* amastigotes. ROS are probably involved in resistance to infection with this species because mice that are unable to activate the respiratory burst by the regular administration of apocynin, an inhibitor of NADPH oxidase, do not control the infection as in untreated animals (our preliminary results). Together, our results suggest that the elimination of *L. guyanensis in vivo* may occur in early infection due to ROS production, before the development of an adaptive T_h_ response.

 There is evidence that peroxynitrite (ONOO^−^) is not involved in the killing of *L. major *[54, 76], but the role of this important oxidant has not been thoroughly explored. In contrast, the production of nitric oxide and ONOO^−^ has been shown during infection with *L. amazonensis *in BALB/c (more susceptible to infection) and C57BL/6 mice (more resistant to infection). The production of nitric oxide *in vivo *was detected as the nitrosyl hemoglobin complex by electron paramagnetic resonance analysis of nitrosyl hemoglobin in blood drawn from mice and in infected footpads at several time points, and ONOO^−^ formation was inferred from immunodetection of nitrotyrosine [101, 102]. C57BL/6 mice presented higher levels of nitrosyl complexes than BALB/c mice at 6 weeks of infection, at which point lesions became chronic in this partially resistant mouse strain. Nitrosyl complexes increased in BALB/c mice, which was dependent on lesion size. iNOS and nitrotyrosine-containing complexes colocalize in lesion macrophages from both mouse strains, and the most probable agent of protein nitration is ONOO^−^ [102]. Peroxynitrite killed *L. amazonensis *axenic amastigotes *in vitro *more efficiently than nitric oxide [102]. The authors proposed that in the susceptible mouse strain, ONOO^−^ is involved in tissue damage. It is possible that the delayed production of ONOO^−^ impairs the capacity of BALB/c mice to control *L. amazonensis*. Treatment of C57BL/6 mice with Tempol, a stable cyclic nitroxide radical that protects cells from damage due to oxidative stress, promoted larger lesions, parasite growth, and lower levels of nitric oxide products and nitrotyrosine [103]. Albeit transient, this effect of Tempol provides further evidence that ONOO^−^ is involved in the control of *L. amazonensis in vivo*.

## 5. Concluding Remarks

The role of reactive oxygen and nitrogen species in killing of *Leishmania* has been the subject of many studies, but there is still much that is not understood. The following questions remain: why do some species of parasites resist oxidative stress? Why do cells that can kill parasites with reactive species harbor live parasites? Is there some attrition when parasites enter neutrophils and macrophages? What is the role of peroxinitrite? What is the reason for the differences in the oxidative responses among different species of parasites? What is the role of reactive oxygen and nitrogen species in the inflammatory response? Collective efforts to fully comprehend the mechanisms that produce disease upon infection with *Leishmania* and the strategies hosts employ to avoid them have been made. However, leishmaniasis persists without safe treatments or effective vaccines. Perhaps the recent attention paid to components of the innate immune system might help to unravel this complex parasite-host relationship. 

## References

[1] Murray HW, Berman JD, Davies CR, Saravia NG (2005). Advances in leishmaniasis. *Lancet*.

[2] Kedzierski L, Zhu Y, Handman E (2006). *Leishmania* vaccines: progress and problems. *Parasitology*.

[3] Bañuls AL, Hide M, Prugnolle F (2007). *Leishmania* and the Leishmaniases: a parasite genetic update and advances in taxonomy, epidemiology and pathogenicity in humans. *Advances in Parasitology*.

[4] Killick-Kendrick R (1990). The life-cycle of *Leishmania* in the sandfly with special reference to the form infective to the vertebrate host. *Annales de Parasitologie Humaine et Comparee*.

[5] Filardy AA, Pires DR, Dosreis GA (2011). Macrophages and neutrophils cooperate in immune responses to *Leishmania* infection. *Cellular and Molecular Life Sciences*.

[6] Peters NC, Sacks DL (2009). The impact of vector-mediated neutrophil recruitment on cutaneous leishmaniasis. *Cellular Microbiology*.

[7] Sacks D, Noben-Trauth N (2002). The immunology of susceptibility and resistance to *Leishmania major* in mice. *Nature Reviews Immunology*.

[8] Horta MF (1997). Pore-forming proteins in pathogenic protozoan parasites. *Trends in Microbiology*.

[9] Noronha FSM, Ramalho-Pinto FJ, Horta MF (1996). Cytolytic activity in the genus *Leishmania*: involvement of a putative pore-forming protein. *Infection and Immunity*.

[10] Noronha FSM, Cruz JS, Beirão PSL, Horta MF (2000). Macrophage damage by *Leishmania amazonensis* cytolysin: evidence of pore formation on cell membrane. *Infection and Immunity*.

[11] Almeida-Campos FR, Horta MF (2000). Proteolytic activation of leishporin: evidence that *Leishmania amazonensis* and *Leishmania guyanensis* have distinct inactive forms. *Molecular and Biochemical Parasitology*.

[12] Castro-Gomes T, Almeida-Campos FR, Calzavara-Silva CE, da Silva RA, Frézard F, Horta MF (2009). Membrane binding requirements for the cytolytic activity of *Leishmania amazonensis* leishporin. *FEBS Letters*.

[13] Noronha FS, Ramalho-Pinto FJ, Horta MF (1994). Identification of a putative pore-forming hemolysin active at acid pH in *Leishmania amazonensis*. *Brazilian Journal of Medical and Biological Research*.

[14] Mosmann TR, Cherwinski H, Bond MW (1986). Two types of murine helper T cell clone. I. Definition according to profiles of lymphokine activities and secreted proteins. *Journal of Immunology*.

[15] Corraliza IM, Soler G, Eichmann K, Modolell M (1995). Arginase induction by suppressors of nitric oxide synthesis (IL-4, IL-10 and PGE2) in murine bone-marrow-derived macrophages. *Biochemical and Biophysical Research Communications*.

[16] Liew FY, Millott S, Parkinson C, Palmer RMJ, Moncada S (1990). Macrophage killing of *Leishmania* parasite in vivo is mediated by nitric oxide from L-arginine. *Journal of Immunology*.

[17] Locksley RM, Heinzel FP, Sadick MD, Holaday BJ, Gardner KD (1987). Murine cutaneous leishmaniasis: susceptibility correlates with differential expansion of helper T-cell subsets. *Annales de l’Institut Pasteur. Immunology*.

[18] Scott P, Natovitz P, Coffman RL, Pearce E, Sher A (1988). Immunoregulation of cutaneous leishmaniasis. T cell lines that transfer protective immunity or exacerbation belong to different T helper subsets and respond to distinct parasite antigens. *Journal of Experimental Medicine*.

[19] Green SJ, Meltzer MS, Hibbs JB, Nacy CA (1990). Activated macrophages destroy intracellular *Leishmania major* amastigotes by an L-arginine-dependent killing mechanism. *Journal of Immunology*.

[20] Soong L, Chang CH, Sun J (1997). Role of CD4+ T cells in pathogenesis associated with *Leishmania amazonensis* infection. *Journal of Immunology*.

[21] Vanloubbeeck YF, Ramer AE, Jie F, Jones DE (2004). CD4+ Th1 cells induced by dendritic cell-based immunotherapy in mice chronically infected with *Leishmania amazonensis* do not promote healing. *Infection and Immunity*.

[22] Qi H, Ji J, Wanasen N, Soong L (2004). Enhanced replication of *Leishmania amazonensis* amastigotes in gamma interferon-stimulated murine macrophages: implications for the pathogenesis of cutaneous leishmaniasis. *Infection and Immunity*.

[23] Vanloubbeeck Y, Jones DE (2004). Protection of C3HeB/FeJ mice against *Leishmania amazonensis* challenge after previous *Leishmania major* infection. *American Journal of Tropical Medicine and Hygiene*.

[24] González-Lombana CZ, Santiago HC, Macedo JP (2008). Early infection with *Leishmania major* restrains pathogenic response to *Leishmania amazonensis* and parasite growth. *Acta Tropica*.

[25] Afonso LCC, Scott P (1993). Immune responses associated with susceptibility of C57BL/10 mice to *Leishmania amazonensis*. *Infection and Immunity*.

[26] Guevara-Mendoza O, Une C, Franceschi Carreira P, Örn A (1997). Experimental infection of Balb/c mice with *Leishmania panamensis* and *Leishmania mexicana* induction of early IFN-*γ* but not IL-4 is associated with the development of cutaneous lesions. *Scandinavian Journal of Immunology*.

[27] DeKrey GK, Lima HC, Titus RG (1998). Analysis of the immune responses of mice to infection with *Leishmania* braziliensis. *Infection and Immunity*.

[28] Mahon-Pratt D, Alexander J (2004). Does the *Leishmania major* paradigm of pathogenesis and protection hold for New World cutaneous leishmaniases or the visceral disease?. *Immunological Reviews*.

[29] Stafford JL, Neumann NF, Belosevic M (2002). Macrophage-mediated innate host defense against protozoan parasites. *Critical Reviews in Microbiology*.

[30] Scharton TM, Scott P (1993). Natural killer cells are a source of interferon *γ* that drives differentiation of CD4+ T cell subsets and induces early resistance to *Leishmania major* in mice. *Journal of Experimental Medicine*.

[31] Birnbaum R, Craft N (2011). Innate immunity and *Leishmania* vaccination strategies. *Dermatologic Clinics*.

[32] Diefenbach A, Schindler H, Donhauser N (1998). Type 1 interferon (IFN*α*/*β*) and type 2 nitric oxide synthase regulate the innate immune response to a protozoan parasite. *Immunity*.

[33] Bancroft GJ, Schreiber RD, Bosma GC (1987). A T cell-independent mechanism of macrophage activation by interferon-*γ*. *Journal of Immunology*.

[34] Dileepan KN, Simpson KM, Stechschulte DJ (1989). Modulation of macrophage superoxide-induced cytochrome c reduction by mast cells. *Journal of Laboratory and Clinical Medicine*.

[35] Hao J, Wu X, Xia S (2010). Current progress in *γδ* T-cell biology. *Cellular and Molecular Immunology*.

[36] Munder M, Mallo M, Eichmann K, Modolell M (1998). Murine macrophages secrete interferon *γ* upon combined stimulation with interleukin (IL)-12 and IL-18: a novel pathway of autocrine macrophage activation. *Journal of Experimental Medicine*.

[37] Puddu P, Carollo M, Pietraforte I (2005). IL-2 induces expression and secretion of IFN-*γ* in murine peritoneal macrophages. *Journal of Leukocyte Biology*.

[38] Fujii S, Motohashi S, Shimizu K, Nakayama T, Yoshiga Y, Taniguchi M (2010). Adjuvant activity mediated by iNKT cells. *Seminars in Immunology*.

[39] van Assche T, Deschacht M, Da Luz RAI, Maes L, Cos P (2011). *Leishmania*-macrophage interactions: insights into the redox biology. *Free Radical Biology and Medicine*.

[40] Ogier-Denis E, Ogier-Denis SB, Vandewalle A (2008). NOX enzymes and Toll-like receptor signaling. *Seminars in Immunopathology*.

[41] Chen GY, Nuñez G (2010). Sterile inflammation: sensing and reacting to damage. *Nature Reviews Immunology*.

[42] Carta S, Castellani P, Delfino L, Tassi S, Venè R, Rubartelli A (2009). DAMPs and inflammatory processes: the role of redox in the different outcomes. *Journal of Leukocyte Biology*.

[43] Nauseef WM (2007). How human neutrophils kill and degrade microbes: an integrated view. *Immunological Reviews*.

[44] Babior BM (1999). NADPH oxidase: an update. *Blood*.

[45] Mizrahi A, Berdichevsky Y, Ugolev Y (2006). Assembly of the phagocyte NADPH oxidase complex: chimeric constructs derived from the cytosolic components as tools for exploring structure-function relationships. *Journal of Leukocyte Biology*.

[46] Hampton MB, Kettle AJ, Winterbourn CC (1998). Inside the neutrophil phagosome: oxidants, myeloperoxidase, and bacterial killing. *Blood*.

[47] Nathan C, Shiloh MU (2000). Reactive oxygen and nitrogen intermediates in the relationship between mammalian hosts and microbial pathogens. *Proceedings of the National Academy of Sciences of the United States of America*.

[48] Radi R, Peluffo G, Alvarez MN, Naviliat M, Cayota A (2001). Unraveling peroxynitrite formation in biological systems. *Free Radical Biology and Medicine*.

[49] Peters NC, Egen JG, Secundino N (2008). In vivo imaging reveals an essential role for neutrophils in leishmaniasis transmitted by sand flies. *Science*.

[50] Beil WJ, Meinardus-Hager G, Neugebauer DC, Sorg C (1992). Differences in the onset of the inflammatory response to cutaneous leishmaniasis in resistant and susceptible mice. *Journal of Leukocyte Biology*.

[51] Nussler AK, Billiar TR (1993). Inflammation, immunoregulation, and inducible nitric oxide synthase. *Journal of Leukocyte Biology*.

[52] Segal AW (2005). How neutrophils kill microbes. *Annual Review of Immunology*.

[53] Charmoy M, Megnekou R, Allenbach C (2007). *Leishmania major* induces distinct neutrophil phenotypes in mice that are resistant or susceptible to infection. *Journal of Leukocyte Biology*.

[54] Blos M, Schleicher U, Rocha FJS, Meißner U, Röllinghoff M, Bogdan C (2003). Organ-specific and stage-dependent control of *Leishmania major* infection by inducible nitric oxide synthase and phagocyte NADPH oxidase. *European Journal of Immunology*.

[55] Mallinson DJ, Lackie JM, Coombs GH (1989). The oxidative response of rabbit peritoneal neutrophils to leishmanias and other trypanosomatids. *International Journal for Parasitology*.

[56] Laufs H, Müller K, Fleischer J (2002). Intracellular survival of *Leishmania major* in neutrophil granulocytes after uptake in the absence of heat-labile serum factors. *Infection and Immunity*.

[57] Mollinedo F, Janssen H, De La Iglesia-Vicente J, Villa-Pulgarin JA, Calafat J (2010). Selective fusion of azurophilic granules with *Leishmania*-containing phagosomes in human neutrophils. *Journal of Biological Chemistry*.

[58] Bisti S, Konidou G, Boelaert J, Lebastard M, Soteriadou K (2006). The prevention of the growth of *Leishmania major* progeny in BALB/c iron-loaded mice: a process coupled to increased oxidative burst, the amplitude and duration of which depend on initial parasite developmental stage and dose. *Microbes and Infection*.

[59] Bisti S, Soteriadou K (2006). Is the reactive oxygen species-dependent-NF-*κ*B activation observed in iron-loaded BALB/c mice a key process preventing growth of *Leishmania major* progeny and tissue-damage?. *Microbes and Infection*.

[60] Ribeiro-Gomes FL, Moniz-de-Souza MCA, Alexandre-Moreira MS (2007). Neutrophils activate macrophages for intracellular killing of *Leishmania major* through recruitment of TLR4 by neutrophil elastase. *Journal of Immunology*.

[61] Ribeiro-Gomes FL, Otero AC, Gomes NA (2004). Macrophage Interactions with Neutrophils Regulate *Leishmania major* Infection. *Journal of Immunology*.

[62] Afonso L, Borges VM, Cruz H (2008). Interactions with apoptotic but not with necrotic neutrophils increase parasite burden in human macrophages infected with *Leishmania amazonensis*. *Journal of Leukocyte Biology*.

[63] Novais FO, Santiago RC, Bafica A (2009). Neutrophils and macrophages cooperate in host resistance against *Leishmania braziliensis* infection. *Journal of Immunology*.

[64] de Souza Carmo EV, Katz S, Barbiéri CL (2010). Neutrophils reduce the parasite burden in *Leishmania (Leishmania) amazonensis* macrophages. *PLoS ONE*.

[65] Brinkmann V, Reichard U, Goosmann C (2004). Neutrophil extracellular traps kill bacteria. *Science*.

[66] Papayannopoulos V, Zychlinsky A (2009). NETs: a new strategy for using old weapons. *Trends in Immunology*.

[67] Guimarães-Costa AB, Nascimento MTC, Froment GS (2009). *Leishmania amazonensis* promastigotes induce and are killed by neutrophil extracellular traps. *Proceedings of the National Academy of Sciences of the United States of America*.

[68] Fuchs TA, Abed U, Goosmann C (2007). Novel cell death program leads to neutrophil extracellular traps. *Journal of Cell Biology*.

[69] Papayannopoulos V, Metzler KD, Hakkim A, Zychlinsky A (2010). Neutrophil elastase and myeloperoxidase regulate the formation of neutrophil extracellular traps. *Journal of Cell Biology*.

[70] Ritter U, Frischknecht F, van Zandbergen G (2009). Are neutrophils important host cells for *Leishmania* parasites?. *Trends in Parasitology*.

[71] Bogdan C, Röllinghoff M (1998). The immune response to *Leishmania*: mechanisms of parasite control and evasion. *International Journal for Parasitology*.

[72] Piani A, Ilg T, Elefanty AG, Curtis J, Handman E (1999). *Leishmania major* proteophosphoglycan is expressed by amastigotes and has an immunomodulatory effect on macrophage function. *Microbes and Infection*.

[73] Kavoosi G, Ardestani SK, Kariminia A, Tavakoli Z (2006). Production of nitric oxide by murine macrophages induced by lipophosphoglycan of *Leishmania major*. *The Korean Journal of Parasitology*.

[74] Green SJ, Crawford RM, Hockmeyer JT, Meltzer MS, Nacy CA (1990). *Leishmania major* amastigotes initiate the L-arginine-dependent killing mechanism in IFN-*γ*-stimulated macrophages by induction of tumor necrosis factor-*α*1. *Journal of Immunology*.

[75] Wei XQ, Charles IG, Smith A (1995). Altered immune responses in mice lacking inducible nitric oxide synthase. *Nature*.

[76] Assreuy J, Cunha FQ, Epperlein M (1994). Production of nitric oxide and superoxide by activated macrophages and killing of *Leishmania major*. *European Journal of Immunology*.

[77] Wei XQ, Leung BP, Niedbala W (1999). Altered immune responses and susceptibility to *Leishmania major* and Staphylococcus aureus infection in IL-18-deficient mice. *Journal of Immunology*.

[78] Ji J, Sun J, Soong L (2003). Impaired expression of inflammatory cytokines and chemokines at early stages of infection with *Leishmania amazonensis*. *Infection and Immunity*.

[79] Gomes IN, Calabrich AF, Tavares RS, Wietzerbin J, Rodrigues De Freitas LA, Tavares Veras PS (2003). Differential properties of CBA/J mononuclear phagocytes recovered from an inflammatory site and probed with two different species of *Leishmania*. *Microbes and Infection*.

[80] Giorgio S, Linares E, Ischiropoulos H, Von Zuben FJ, Yamada A, Augusto O (1998). In vivo formation of electron paramagnetic resonance-detectable nitric oxide and of nitrotyrosine is not impaired during murine leishmaniasis. *Infection and Immunity*.

[81] Liew FY, Li Y, Moss D, Parkinson C, Rogers MV, Moncada S (1991). Resistance to *Leishmania major* infection correlates with the induction of nitric oxide synthase in murine macrophages. *European Journal of Immunology*.

[82] Stenger S, Thüring H, Röllinghoff M, Bogdan C (1994). Tissue expression of inducible nitric oxide synthase is closely associated with resistance to *Leishmania major*. *Journal of Experimental Medicine*.

[83] Zidek Z, Frankova D, Boubelik M (2000). Genetic variation in in-vitro cytokine-induced production of nitric oxide by murine peritoneal macrophages. *Pharmacogenetics*.

[84] Santos JL, Andrade AA, Dias AAM (2006). Differential sensitivity of C57BL/6 (M-1) and BALB/c (M-2) macrophages to the stimuli of IFN-*γ*/LPS for the production of NO: correlation with iNOS mRNA and protein expression. *Journal of Interferon and Cytokine Research*.

[85] Lima-Santos J, Jardim IS, Teixeira SM, Horta MF (1999). Diffferential induction of nitric oxide synthase in C57BL/6 and BALB/c macrophages by IFN-gamma and TNF-alpha or LPS. *Abstracts of the Brazilian Society of Immunolgy Meeting*.

[86] Dileepan KN, Page JC, Li Y, Stechschulte DJ (1995). Direct activation of murine peritoneal macrophages for nitric oxide production and tumor cell killing by interferon-*γ*. *Journal of Interferon and Cytokine Research*.

[87] Oswald IP, Afroun S, Bray D, Petit JF, Lemaire G (1992). Low response of BALB/c macrophages to priming and activating signals. *Journal of Leukocyte Biology*.

[88] Jardim IS, Santos JL, Horta MF (1999). Differential production of nitric oxide by murine macrophages from *Leishmania* resistant and susceptible mice strains. *Memórias do Instituto Oswaldo Cruz*.

[89] Jardim IS, Santos JL, Horta MF, Ramalho-Pinto FJ (2011). Inhibition of the production of nitric oxide impairs cytotoxicity of macrophages to *Leishmania amazonensis*. *Memórias do Instituto Oswaldo Cruz*.

[90] Mills CD, Kincaid K, Alt JM, Heilman MJ, Hill AM (2000). M-1/M-2 macrophages and the Th1/Th2 paradigm. *Journal of Immunology*.

[91] Giudice A, Camada I, Leopoldo PTG (2007). Resistance of *Leishmania (Leishmania) amazonensis and Leishmania (Viannia) braziliensis* to nitric oxide correlates with disease severity in Tegumentary Leishmaniasis. *BMC Infectious Diseases*.

[92] Babior BM (2004). NADPH oxidase. *Current Opinion in Immunology*.

[93] Laroux S, Romero X, Wetzler L, Engel P, Terhorst C (2005). Cutting edge: MyD88 controls phagocyte NADPH oxidase function and killing of gram-negative bacteria. *Journal of Immunology*.

[94] Pollock JD, Williams DA, Gifford MAC (1995). Mouse model of X-linked chronic granulomatous disease, an inherited defect in phagocyte superoxide production. *Nature Genetics*.

[95] Degrossoli A, Arrais-Silva WW, Colhone MC, Gadelha FR, Joazeiro PP, Giorgio S (2011). The influence of low oxygen on macrophage response to *Leishmania* infection. *Scandinavian Journal of Immunology*.

[96] Mukbel RM, Patten C, Gibson K, Ghosh M, Petersen C, Jones DE (2007). Macrophage killing of *Leishmania amazonensis* amastigotes requires both nitric oxide and superoxide. *American Journal of Tropical Medicine and Hygiene*.

[97] Khouri R, Bafica A, Silva MDPP (2009). IFN-*β* impairs superoxide-dependent parasite killing in human macrophages: evidence for a deleterious role of SOD1 in cutaneous leishmaniasis. *Journal of Immunology*.

[98] Neal RA, Hale C (1983). A comparative study of susceptibility of inbred and outbred mouse strains compared with hamsters to infection with New World cutaneous leishmaniases. *Parasitology*.

[99] Sousa-Franco J, Araujo-Mendes E, Silva-Jardim I (2006). Infection-induced respiratory burst in BALB/c macrophages kills *Leishmania guyanensis* amastigotes through apoptosis: possible involvement in resistance to cutaneous leishmaniasis. *Microbes and Infection*.

[100] Scott P, Sher A (1986). A spectrum in the susceptibility of Leishmanial strains to intracellular killing by murine macrophages. *Journal of Immunology*.

[101] Giorgio S, Linares E, Capurro MDL, De Bianchi AG, Augusto O (1996). Formation of nitrosyl hemoglobin and nitrotyrosine during murine leishmaniasis. *Photochemistry and Photobiology*.

[102] Linares E, Giorgio S, Mortara RA, Santos CXC, Yamada AT, Augusto O (2001). Role of peroxynitrite in macrophage microbicidal mechanisms in vivo revealed by protein nitration and hydroxylation. *Free Radical Biology and Medicine*.

[103] Linares E, Giorgio S, Augusto O (2008). Inhibition of in vivo leishmanicidal mechanisms by tempol: nitric oxide down-regulation and oxidant scavenging. *Free Radical Biology and Medicine*.

